# The Use of Ion Milling for Surface Preparation for EBSD Analysis

**DOI:** 10.3390/ma14143970

**Published:** 2021-07-16

**Authors:** Wojciech J. Nowak

**Affiliations:** 1 Department of Materials Science, Faculty of Mechanical Engineering and Aeronautics, Rzeszow University of Technology, al. Powstanców Warszawy 12, 35-959 Rzeszow, Poland; wjnowak@prz.edu.pl; Tel.: +48-506-674-497; 2 Forschungszentrum Julich IEK-2, Leo-Brandt-Straße, 52428 Julich, Germany

**Keywords:** plasma etching, GD-OES, EBSD, alloy microstructure

## Abstract

An electron backscattered diffraction (EBSD) method provides information about the crystallographic structure of materials. However, a surface subjected to analysis needs to be well-prepared. This usually requires following a time-consuming procedure of mechanical polishing. The alternative methods of surface preparation for EBSD are performed via electropolishing or focus ion beam (FIB). In the present study, plasma etching using a glow discharge optical emission spectrometer (GD-OES) was applied for surface preparation for EBSD analysis. The obtained results revealed that plasma etching through GD-OES can be successfully used for surface preparation for EBSD analysis. However, it was also found that the plasma etching is sensitive for the alloy microstructure, i.e., the presence of intermetallic phases and precipitates such as carbides possess a different sputtering rate, resulting in non-uniform plasma etching. Preparation of the cross-section of oxidized CM247 revealed a similar problem with non-uniformity of plasma etching. The carbides and oxide scale possess a lower sputtering rate than the metallic matrix, which caused formation of relief. Based on obtained results, possible resolutions to suppress the effect of different sputtering rates are proposed.

## 1. Introduction

Electron backscattered diffraction (EBSD) is a scanning electron microscope based on an analytical method providing information about the crystallographic orientation of the material [1,2,3]. The effective penetration depths of backscattered electrons depend on electron energy, material density, and, to a lesser extent, crystallographic orientation and typically is less than 10–30 nm [1]. The EBSD method is very sensitive to plastic deformation of the near-surface region. If this deformation is present in the material, then EBSD indexing effectiveness is at a very low level [4]. Thus, sample surface preparation is a crucial step for sample preparation using EBSD analysis. Ideally, a surface for EBSD analysis should be free of plastic deformation and flat. Usually, the samples surfaces for EBSD analysis are prepared by a series of mechanical polishing steps finishing at final polishing followed by vibratory polishing for about 30 min. However, such preparation is relatively time-consuming and there is a danger of introducing a deformation in the near-surface region. Other methods for surface preparation for EBSD analysis are chemical polishing [5,6,7,8], electropolishing [9] or ion milling [10]. Plasma etching [11,12,13] is one of the methods applied for the etching of polymers [14], single crystal diamond [15], SiO_2_ layers [16], carbon [17] or Ti-Al_3_Ti laminates [18]. Usually, low-pressure plasma etching requires costly vacuum equipment [14]. The different surface preparation can affect the results obtained by EBSD [19]. The glow discharge optical emission spectrometry (GD-OES) method is an analytical method that uses a plasma source for material sputtering. However, the main function of the GD-OES is the analysis of the chemical composition of alloys [20,21] or the measurement of depth profiles that show the distribution of the elements content as a function of sputtering time (or depth) [21,22,23]. In the present study, the GD HR Profiler was used as a method for surface preparation prior to EBSD analysis. In this device a sample with a sputtered surface is placed directly into the anode. Thus, no vacuum chamber is necessary. The vacuum is produced directly in the internal part of the anode, and afterward fulfilled by argon with low pressure. Therefore, the procedure for plasma sputtering occurs for no longer than 5 min. A surface plasma etching by GD-OES technique is a fast method providing relatively large area for the analysis. Thus, the GD-OES technique is a promising method for surface preparation for EBSD analysis.

## 2. Materials and Methods

In the present study, a glow discharge optical emission spectrometer (GD-OES) GD-Profiler fabricated by Horiba Jobin Yvon (Paris, France) was used as a tool for sample preparation via plasma etching for electron backscattered diffraction (EBSD) analysis. The used GD-OES device is a GD HR Profiler with RF electrodes. An anode fabricated of copper with the internal diameter of 4 mm was used. Thus, the sputtered area possesses a circle shape with the diameter of 4 mm. In this device argon pressure can easily be controlled and the available pressure range lies between 0 and 1100 Pa. However, it is known that the lowering of the Ar pressure significantly lowers the sputtering rate of metallic material due to the limited number of Ar ions in the plasma able to collide with surface of the material. Thus, an Ar pressure at the level of 300 Pa was adjusted. The studied materials included the steels: 1.4509 (AISI 441) (ThyssenKrupp Nirosta GmbH, Krefeld, Germany) and 1.4521 (AISI 444) (ThyssenKrupp Nirosta GmbH, Krefeld, Germany) and Ni-base superalloy CM247 (Cannon Muskegon Corporation, Muskegon, MI, USA) with the chemical compositions observed in Table 1. The study was divided into two parts: First, the usefulness of the surface preparation by GD-OES was investigated on the steels in as-received conditions. Prior to plasma etching by GD-OES, the surfaces of both steels were ground and polished by the standard metallographic procedure using 1 µm average grain diameter silica suspension for 2 min per step. Then, a series of plasma etchings for different times was applied. The conditions for plasma etching were as follows: argon pressure was 300 MPa while the power was adjusted to 7 W. Plasma etching was performed for 10, 20, 30 and 40 s. After such preparation, the sputtered and non-sputtered regions were investigated by field emission scanning electron microscope (SEM) (Merlin) fabricated by Carl Zeiss (Oerzen, Germany), using energy dispersive X-ray spectroscopy (EDX) (Oxford Instruments, Dresden, Germany) and electron backscattered diffraction (EBSD) detectors (Oxford Instruments, Dresden, Germany). In the second part, an attempt to prepare the cross-sectioned CM247 after air oxidation at 1050 °C for 20 h was performed. During cross-section preparation, an oxidized sample was mounted in conductive thermosetting resin (PolyFast) containing carbon fiber to provide electric conductivity using a hot press (CitoPress-5) fabricated by Struers (Cleveland, OH, USA). The procedure for hot mounting was as follows: A press was heated to 180 °C for 3.5 min using a pressure of 225 bar. After 3.5 min of heating a mounted sample was cooled for 2.5 min. The cross-section was prepared by a series of grinding and polishing steps until final polishing with silica suspension with the average grain diameter of 0.25 µm. Then the cross-section was plasma-etched using the GD-OES device. The argon pressure and power for plasma etching were the same for the steels; however, sputtering time increased to 300 s. After plasma etching the sample was analyzed by EBSD.

## 3. Results

### 3.1. Studies on Steel Surfaces

#### 3.1.1. Steel 1.4509 (AISI 441)

**Figure 1:**
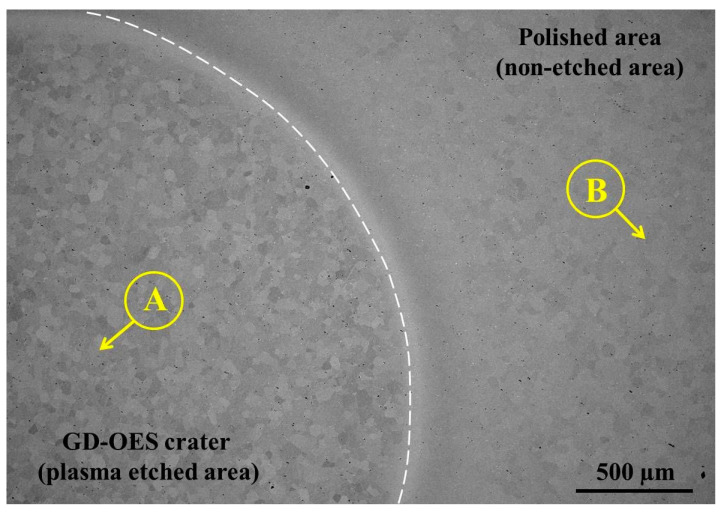
SEM/BSE image of the surface of steel 1.4509 (AISI 441) showing sputtered (**A**) and non-sputtered (**B**) areas.

**Figure 2:**
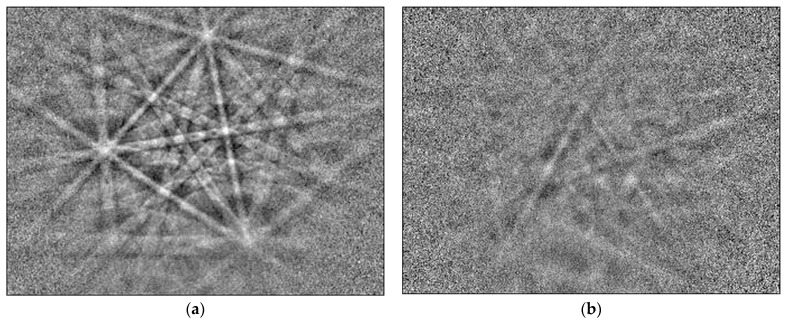
EBSD patterns obtained on: (**a**) sputtered area (crater depth) and (**b**) non-sputtered area (mechanically polished area) of steel 1.4509 (AISI 441).

**Figure 3:**
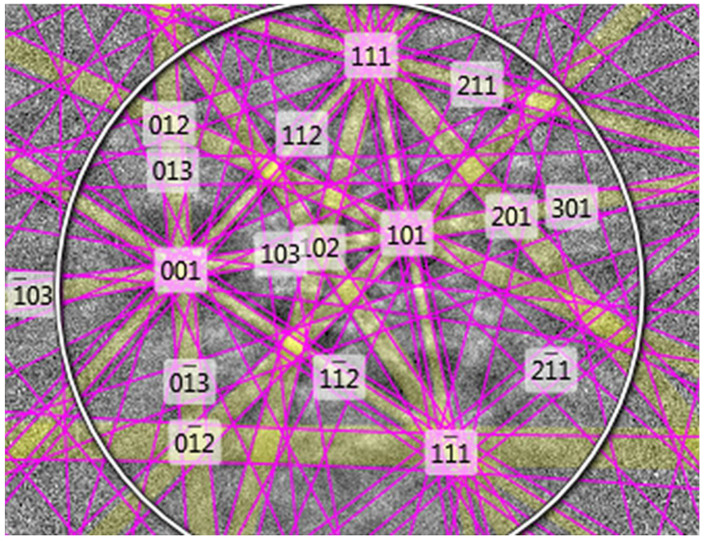
Indexed EBSD Pattern of sputtered area (from Figure 2a) of steel 1.4509 (AISI 441).

To study the potential of plasma etching using the GD-OES device as a fast method of surface preparation for EBSD analysis, flat, mechanically polished specimens of steel were investigated. For plasma etching, an anode with a 4 mm diameter of pure copper was chosen. Plasma etching was performed for 10, 20, 30 and 40 s. EBSD analyses revealed that the best results are obtained for plasma etching for 40 s. Thus, only the results obtained for 40 s plasma etching are shown. Figure 1 shows the SEM/BSE image of the 1.4509 steel after 40 s of plasma sputtering. As marked in the left bottom region, a plasma-etched area is present while upper right corner shows the area after mechanical polishing. A border between these two areas is schematically shown by the white dashed line. Using an anode with 4 mm diameter results in providing 12.56 mm^2^ of plasma-etched area enabled EBSD analysis. Figure 2a shows the EBSD patterns (so-called Kikuchis patterns) obtained on the plasma-etched area. Comparing them to the same patterns measured on the mechanically polished area (Figure 2b) one can clearly see that plasma etching results in providing stronger patterns than simple mechanical polishing. EBSD patterns obtained on plasma-etched regions were easily indexed, as shown in Figure 3.

**Figure 4:**
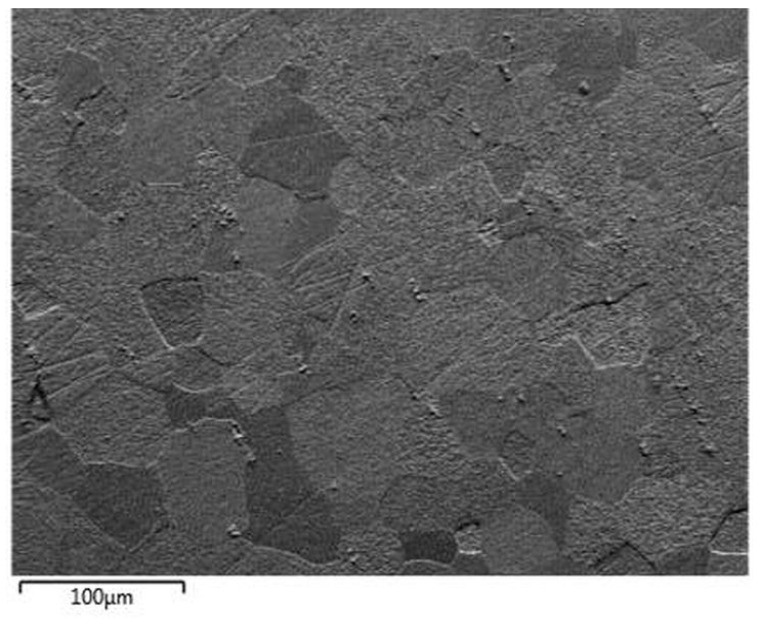
FSD mixed image of sputtered area of steel 1.4509 (AISI 441).

**Figure 5:**
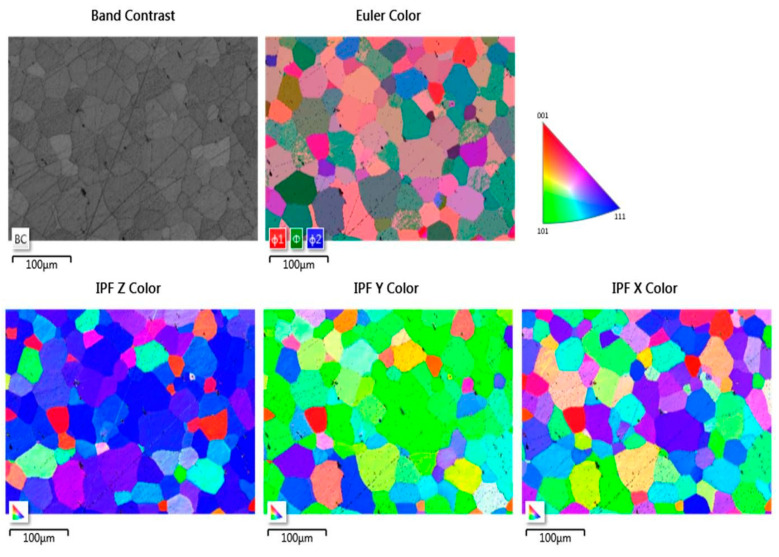
EBSD images of sputtered area of steel 1.4509 (AISI 441).

Figure 3 shows the image of the plasma sputtered surface of steel 1.4509 obtained using a forward scatter detector (FSD). As shown, each grain of the material can be easily distinguished. However, small scratches are still present even after plasma sputtering (see Figure 4 and Figure 5 for band contrast). Nevertheless, the analysis by EBSD was fully possible, as shown on Euler color and inverse pole figures (IPF) (Figure 5). Full identification of each grain was possible.

#### 3.1.2. Steel 1.4521 (AISI 444)

**Figure 6:**
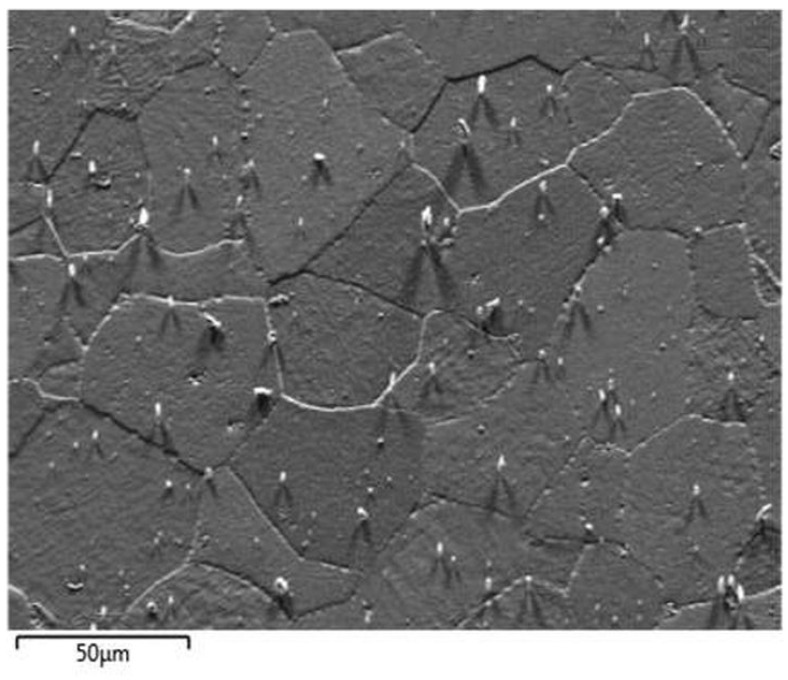
FSD mixed image of sputtered area of steel 1.4521 (AISI 444).

**Figure 7:**
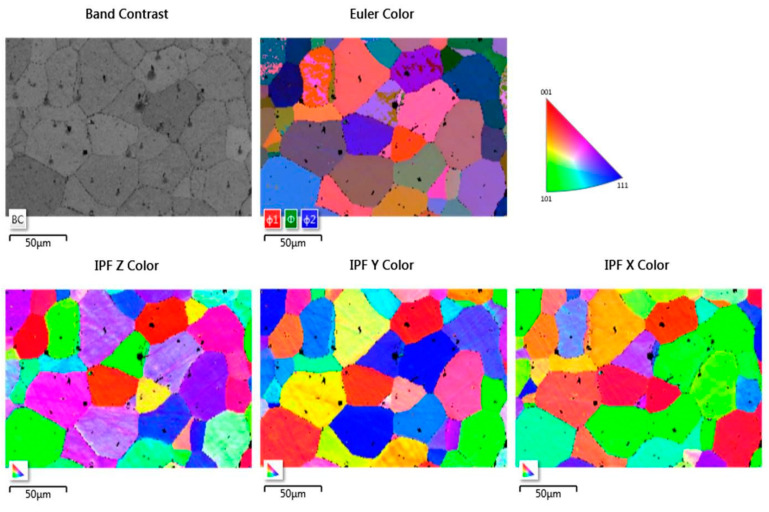
EBSD images of sputtered area of steel 1.4521 (AISI 444).

**Figure 8:**
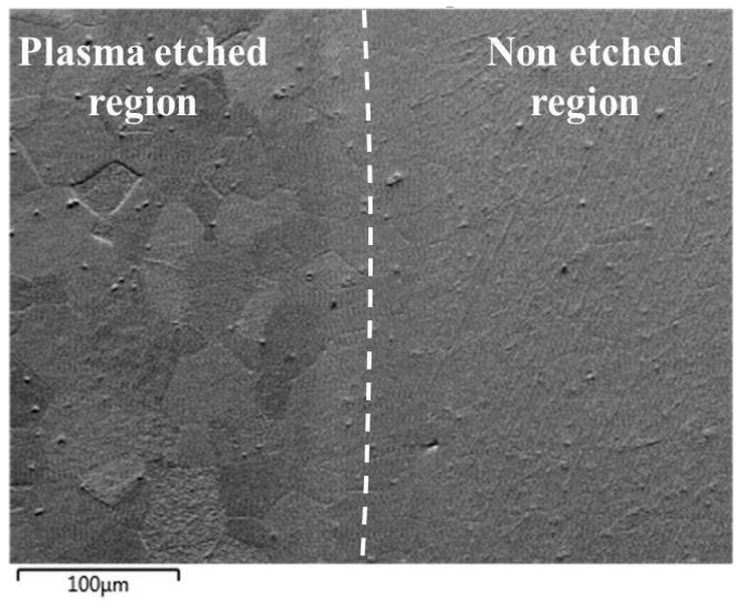
FSD mixed image of the edge between sputtered and non-sputtered area of steel 1.4521 (AISI 444).

**Figure 9:**
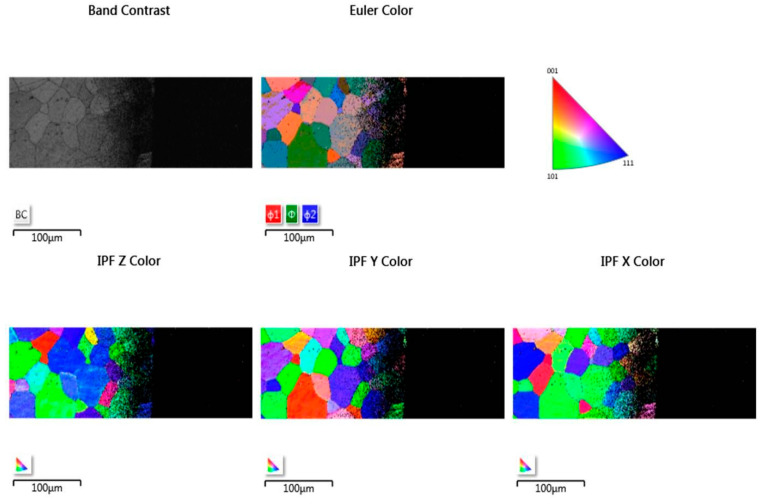
EBSD images of sputtered area of steel 1.4521 (AISI 444) at the sputtered and non-sputtered interface.

The image of the plasma-etched surface of steel 1.4521 obtained using the forward scatter detector (FSD) shown in Figure 6 revealed that the plasma-etching process was also successful. This means that the grains present in the plasma-etched region can clearly be distinguished. However, spiky-shaped intrusions are present within the volume of grains. These precipitates seem to stick out of the 1.4521 steel. Moreover, as shown in Figure 7, the locations of the above-mentioned precipitates are visible as black points on EBSD maps within the grains as well as at the grain boundaries, which means that they are impossible to identify. To illustrate the effectiveness of plasma etching on the quality of surface preparation for the EBSD analysis, the measurement was performed at the interface between the etched area by GD-OES sputtering and non-etched area (Figure 8). In the etched region, grains are more clearly visible than in the non-etched area. The consequence of the latter is that EBSD analysis is not effective in the non-sputtered region while such analysis is fully possible in the sputtered region (Figure 9). However, Figure 9 reveals that very close to the crater edge, a narrow region exists in which the grain orientation determination is disturbed (region close to the middle of Euler and IPF images in Figure 9).

### 3.2. Studies on Cross-Section

**Figure 10:**
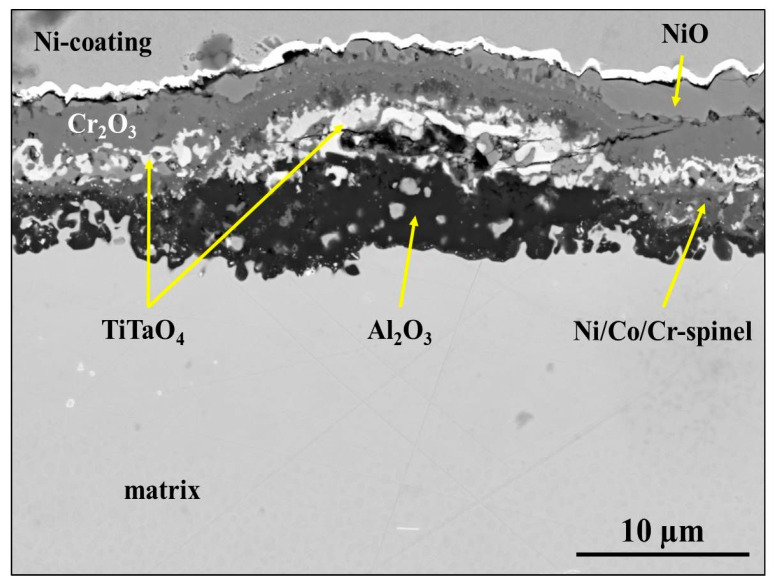
SEM/BSE image of the cross-section of CM247 after air oxidation at 1050 °C for 20 h.

**Figure 11:**
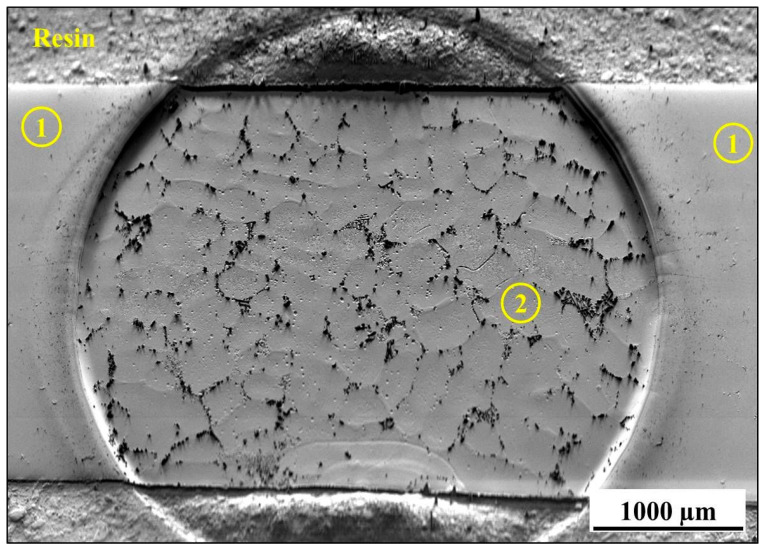
SEM/BSE image of plasma-etched cross-section of CM247 after air oxidation at 1050 °C for 20 h. Region 1 describes the original surface of the cross-section while region 2 shows the plasma-etched region (GD-OES crater bottom).

**Figure 12:**
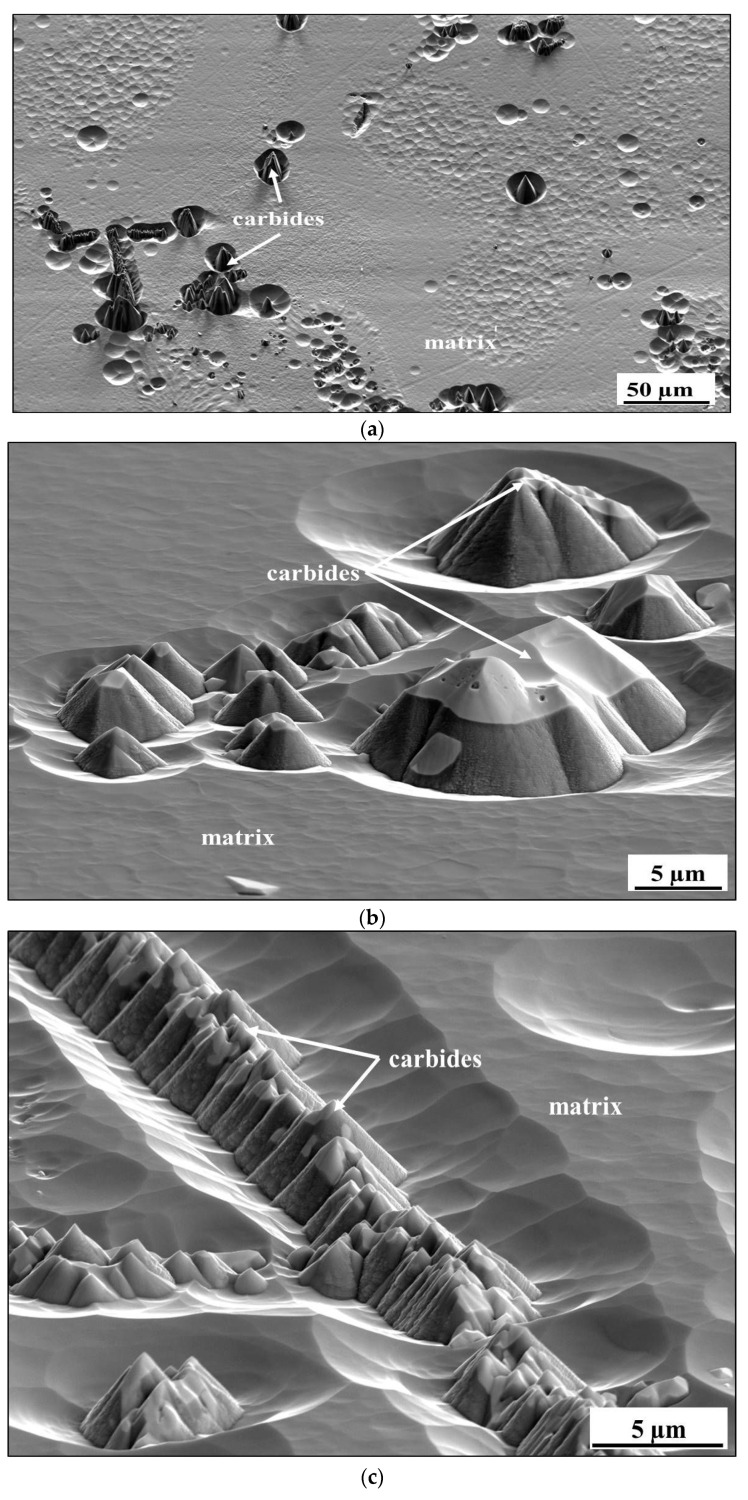
SEM/BSE images of the bottom of the sputtered area on the cross-section of CM247 after air oxidation at 1050 °C for 20 h showing: (**a**) an overview of the crater bottom, (**b**) single carbides and (**c**) carbides located at the grain boundaries of CM247.

**Figure 13:**
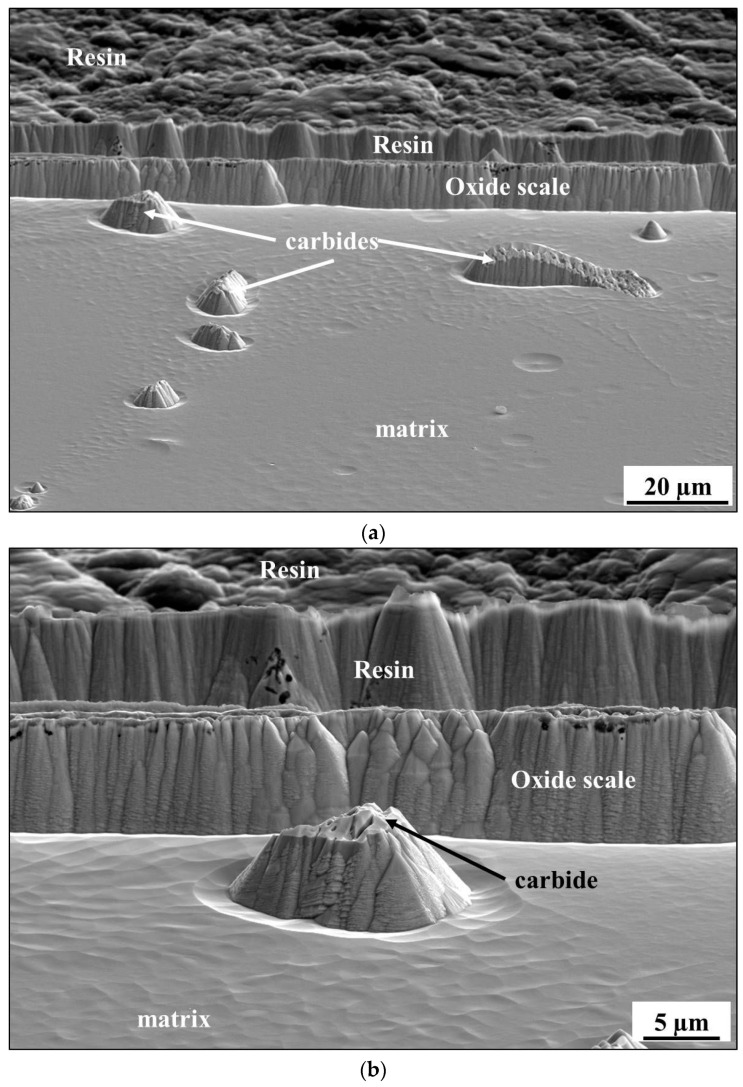
SEM/BSE images showing the sputtered area on the cross-section of CM247 after air oxidation at 1050 °C for 20 h at the base alloy/oxide scale interface taken at: (**a**) low and (**b**) high magnification.

After validation of successful surface preparation by GD-OES on flat metallic surfaces, we attempted to prepare the cross-section of CM247 after oxidation. The microstructure of CM247 after air oxidation at 1050 °C for 20 h is shown in Figure 10. The SEM/BSE image of the cross-section revealed that CM247 formed a multilayered oxide scale on its surface. The oxide scale consisted of NiO in the outer part below which Cr_2_O_3_ is present. In the middle part of the oxide scale TiTaO_4_ particles and Ni/Co/Cr-spinel formed, and at the oxide scale/alloy interface Al_2_O_3_ formed. The cross-section of the sample was subjected to preparation for EBSD analysis by GD-OES plasma sputtering. The obtained crater is shown in Figure 11, where regions marked as 1 represent non-etched regions and region 2 is a plasma-etched region (inside the GD-OES crater). Even the metallic matrix was not equally etched (Figure 11): dark precipitates aligned the most along the grain boundaries. Images of the region at the crater bottom captured at higher magnification revealed that non-uniform sputtering occurred near the single carbides precipitates (Figure 12a,b) and also near the carbides aligned along the grain boundary (Figure 12c). Observation of the region near the alloy/oxide scale interface showed that not only carbides caused non-uniform sputtering, but also the oxide scale was sputtered slower than the metallic alloy (Figure 13a,b). As the result a significant difference between the levels of resin, oxide scale and the alloy was observed (see e.g., Figure 13b). The main focus of plasma etching the cross-section was to prepare both regions, the oxide scale and metallic matrix. This was not successful, so no EBSD analysis was performed on the prepared cross-section of the oxidized CM-247.

## 4. Discussion

The results obtained on flat, mechanically polished, metallic samples clearly show that a surface etching by plasma sputtering using the GD-OES device can successfully be applied as a method for fast preparation of a surface for EBSD analysis. However, plasma etching was sensitive for the alloy microstructure and phase composition. As described by Niewolak et al. [24], steel 1.4509 contains a small amount of Ti-rich carbonitrides, while in the microstructure of steel 1.4521 an intermetallic σ-FeCr phase is found. Moreover, this phase was uniformly distributed over the alloy volume. As stated in the literature, different phases are characterized by different sputtering rates [25,26], which causes a difference in the heights of precipitates after sputtering. As shown in the present study, σ-FeCr possesses a slower sputtering rate than the matrix; thus, the surface of steel 1.4521 was etched non-uniformly. As the result of the latter, precipitates of σ-FeCr were impossible to identify through EBSD analysis. A similar situation occurred in the preparation of the cross-section of CM247 after oxidation. A microstructure of CM247 consists of Ti/W/Ta/Hf-containing primary carbide precipitates that accumulated mainly at the grain boundaries and/or in the interdendritic (eutectic) regions [27]. These carbides aligned along grain boundaries caused an occurrence of relief near the grain boundaries. Moreover, the difference between the sputtering rate of the alloys and the formed oxide scale is clearly visible. The whole oxide scale was sputtered slower than the alloy; thus, in the images an oxide scale is present as a “shelf” placed more than 5 µm above the level of etched metallic matrix. These observations showed the EBSD analysis is impossible on a sputtered cross-section. To overcome this issue, following possibilities are proposed:

- Shortening of plasma etching time;
- Lowering the plasma parameters, e.g., argon pressure;
- Using so-called “pulse mode” to lower the sputtering rate.

All proposed solutions are the topic of ongoing work.

## 5. Conclusions

Based on the results obtained in the present study, the following conclusions can be drawn:

- Plasma etching by GD-OES is a very fast method for surface preparation for EBSD analysis of homogeneous metallic materials;
- The plasma etching is sensitive to the presence of intermetallic phases as well as carbides due to a large difference in their sputtering rates;
- Preparation of oxidized CM247 by plasma etching in GD-OES failed due to the difference in sputtering rate of the oxide scale and metallic matrix;

Surface preparation by plasma etching using GD-OES is limited to the preparation of metallic materials without secondary phases such as oxides, carbides etc.

## Figures and Tables

**Table 1:** Chemical composition of studied materials in wt %.

Alloy	Chemical Composition wt %												
	Fe	Cr	Ni	Mn	Mo	Nb	Si	Al.	Ti	Ta	Co	W	Hf
1.4509	Bal.	17.4	0.13	0.23	1.6	0.6	0.41	0.03	0.17	-	-	-	-
1.4521	Bal.	17.4	0.24	0.46	0.01	1.3	0.12	0.02	0.09	-	-	-	-
CM247	-	8.2	Bal.	-	0.5	-	-	5.4	0.7	3.4	9.4	9.8	1.3

## Data Availability

The data presented in this study are available on reasonable request from the corresponding author.
